# Locked Plate Fixation of Proximal Humerus Fractures in Non-elderly Adults: Analysis of Short-Term Outcomes

**DOI:** 10.7759/cureus.112925

**Published:** 2026-07-18

**Authors:** Fred Vilson, Yan Fei Wang, Olivia Puhalla, Keila Cortes, John Gentile, Adam Hoffman

**Affiliations:** 1 Department of Orthopedic Surgery, St. Elizabeth Youngstown Hospital, Youngstown, USA; 2 Medical Education, Lake Erie College of Osteopathic Medicine, Erie, USA; 3 Medical Education, Youngstown State University, Youngstown, USA; 4 Medical Education, The Ohio State University, Columbus, USA; 5 Department of Orthopedic Trauma, St. Elizabeth Youngstown Hospital, Youngstown, USA

**Keywords:** interim outcomes, locking plate fixation, non-elderly patients, open reduction internal fixation, proximal humerus fracture

## Abstract

Introduction

Proximal humerus fractures (PHFs) represent the third most common adult fracture, increasingly affecting women and middle-aged individuals. While older patients typically sustain PHFs via low-energy trauma, younger populations more frequently incur these injuries through high-energy mechanisms. Surgical decision-making is influenced by patient-specific factors, with open reduction and internal fixation (ORIF) using locking plates considered a reliable technique for select cases. Despite demonstrated utility, limited data exist regarding functional outcomes of locking plate fixation in non-elderly adults.

Methods

This was a retrospective study of 17 non-elderly patients between the ages of 18 and 65 who underwent ORIF with locking plates for PHFs at a single Level 1 trauma center between January 2014 and December 2024. Patient demographics, fracture classification, comorbidities, and radiographic features were documented. Validated functional outcome measures were collected via phone following informed consent. Subgroup analysis evaluated differences by sex, smoking status, and fracture type.

Results

Revision surgery occurred in two patients, and most demonstrated satisfactory shoulder motion at final follow‑up, aside from one four‑part fracture case with persistent rotational limitation. Functional outcomes and range of motion did not reveal clear differences by sex, smoking status, or fracture classification, though the small cohort limits definitive conclusions.

Conclusion

Although constrained by a small cohort and short‑term follow‑up, our results indicate that ORIF with locking plates may provide acceptable early outcomes for two‑ and three‑part fractures in non‑elderly patients. However, given that only two individuals had four‑part fractures and one continued to exhibit rotational deficits, the data are insufficient to support any conclusions about the effectiveness of ORIF for four‑part injuries. Future studies with greater power are required to better define the role of fracture complexity and patient‑level variables in short- and long‑term recovery.

## Introduction

Proximal humerus fractures (PHFs) are the third most common fractures in adults, with increasing incidence, particularly among women [1]. Older individuals typically experience PHFs due to low-energy trauma, such as falls, which are exacerbated by osteoporosis and reduced strength. Younger patients, however, often sustain PHFs from high-energy trauma, including motor vehicle accidents and occupational hazards [2,3]. Additionally, high-velocity sporting injuries contribute to complex fractures, while middle-aged individuals increasingly present with severe PHFs [4].

Fracture classification commonly relies on the Neer system, which evaluates displacement across four anatomical regions [1]. The AO classification further refines PHF categorization, distinguishing fractures by articular involvement and structural complexity into three major types and 27 subgroups [1,5]. However, Neer and AO classifications have demonstrated low reliability, prompting adoption of Orthopaedic Trauma Association (OTA)/AO for improved clinical utility [4-6].

Treatment decisions consider comorbidities, bone mineral density, lifestyle, and functional demands [3,6]. Most PHFs are managed non-operatively through immobilization, rehabilitation, and pain management, although outcomes can be suboptimal [3,6]. Some surgeons advocate for open reduction and internal fixation (ORIF) in select cases [4]. Locking plate fixation remains a preferred technique for stability, though complications, including axillary nerve injury, are notable [3,6]. Older patients face higher rates of postoperative failure, with ORIF-linked complication rates reaching 44% and failure rates 34% [7]. Despite these risks, PHF management with locking plates has yielded favorable results, with studies reporting up to 80% good-to-excellent outcomes based on validated scoring systems [4]. Given the limited existing data on the use of locking plate constructs for PHFs in non‑elderly individuals, this study seeks to add to the current literature by evaluating short‑term clinical outcomes in this population. Our goal is to provide preliminary insight into the effectiveness of this treatment approach in restoring function and stability.

When analyzing ORIF for PHFs in non-elderly individuals, we asked: What are the reoperation rates and postoperative complications following ORIF for PHFs in non-elderly patients? Are there significant differences in functional outcome scores (American Shoulder and Elbow Surgeons (ASES) [8], Disabilities of the Arm, Shoulder and Hand (DASH) [9], Single Assessment Numeric Evaluation (SANE) [10], and satisfaction) based on patient factors such as sex, smoking status, and fracture classification? Does range of motion differ between Neer fracture classification following ORIF for PHFs in non-elderly individuals?

## Materials and methods

Study design and setting

The Institutional Review Board reviewed this study and determined it to be exempt. This study was designed as a retrospective cohort analysis of orthopedic patients treated at a single Level I trauma center in Northeast Ohio.

Participants

All patients who sustained a PHF and underwent surgical management (CPT 23615) at a single Level I trauma center between January 2014 and December 2024 were screened for eligibility. Procedures were performed by multiple fellowship‑trained orthopedic surgeons. Inclusion criteria consisted of patients treated with ORIF using a locking plate construct.

Patients were excluded if they were younger than 18 years or older than 65 years, if they underwent primary shoulder arthroplasty (hemiarthroplasty, reverse, or anatomic total shoulder arthroplasty) for management of the fracture, or if they had insufficient clinical documentation to confirm fracture pattern or operative details. Additional exclusions included pathologic fractures, periprosthetic fractures, polytrauma patients in whom functional assessment could not be reliably attributed to the proximal humerus injury, and patients lost to follow-up prior to outcome assessment. A total of 17 patients met inclusion criteria and were evaluated for functional outcomes. Three patients were subsequently lost to follow‑up because of incomplete medical records, resulting in 14 patients with available range‑of‑motion data.

Description of experiments, treatment, and surgery

Each patient underwent an initial evaluation, including plain radiographs, which, along with a complete clinical assessment, confirmed the indication for operative intervention. Informed consent for research and questionnaire were obtained via phone. All 17 patients were treated using a standard delto-pectoral approach, utilizing the internervous plane between the deltoid and pectoralis major muscles. Fractures were adequately reduced under direct visualization and stabilized with a locking plate and screw construct. No intra-operative complications were reported across the 17 cases reviewed.

Variables

A thorough chart review was performed, recording and analyzing demographic data such as age, and sex, along with comorbidities including smoking history, diabetes, and osteoporosis. Radiographic data, including fracture patterns was documented. Additionally, information on subsequent procedures involving the index shoulder and all clinical and radiographic follow-up data were collected and analyzed.

Outcome measures and data sources

Functional outcomes questionnaires were administered by phone following verbal informed consent for research to all patients. Functional outcomes were assessed using validated measures, including the DASH score, the ASES Shoulder Score, the SANE, and patient satisfaction surveys. Patients who underwent reoperations on the index shoulder were also asked to complete these outcome measures.

After all data points were collected, a subgroup analysis was completed to determine which known factors may have affected the functional outcome measures and reoperation rates. Several variables were compared including sex, smoking history, history of osteoporosis and fracture classification. 

Bias

This study was subject to several inherent sources of bias. As a retrospective cohort analysis, treatment allocation was not randomized, introducing selection bias based on surgeon preference and patient factors. Clinical and radiographic data were obtained through retrospective chart review, which may underreport complications or suboptimal outcomes. Functional outcome measures (DASH, ASES, and SANE) were collected by telephone after obtaining informed consent; however, limited follow-up precluded consistent in‑person examinations. Consequently, functional results may be biased toward patients who were reachable, willing, and physically able to participate, introducing both response and recall bias. Reliance on patient self‑report may also favor individuals with comparatively better outcomes. Additionally, all calls were conducted by an orthopedic physician, which may have influenced patient responses through interviewer or social desirability bias.

Statistical analysis

Demographics and clinical characteristics were reported as mean and standard deviation (SD) for continuous data or frequency and percentage for categorical data. Descriptive statistics were reported as median and interquartile range. Normality was evaluated with the Shapiro-Wilk test. Given the non‑normal distribution of the data and the very small subgroup sample sizes, analyses of ASES, DASH, and SANE scores were conducted using the Mann-Whitney U test and interpreted as exploratory. An additional subgroup analysis was conducted comparing the functional outcome scores, abduction, and forward flexion of patients with different Neer classification using the Kruskal-Wallis test. Statistical analyses were conducted using native functions in Microsoft Excel (Microsoft, Redmond, WA, USA). Data was ranked, and the U statistic, Z statistics, and H statistics were calculated. Due to the presence of tied ranks, the U statistics were converted into Z statistic using normal approximation. The H statistic was evaluated against a Chi-square distribution. The p-value was derived from the Z statistic and H statistic, with a p-value of < 0.05 considered statistically significant.

## Results

A total of 25 eligible patients were identified, and 17 met all inclusion criteria for final analysis. Most patients in the cohort were female, and the average age was in the early 50s. Follow-up information was available for the majority of patients, with only a few lost due to incomplete records. Across the cohort, revision surgery was uncommon, occurring in only two individuals. At final follow-up, most patients demonstrated satisfactory shoulder motion, although one patient with a four-part fracture continued to experience limitations in rotation. Demographic and clinical characteristics are summarized in Table 1, and revision outcomes and range of motion findings are presented in Table 2.

**Table 1:** Demographics and Clinical Characteristics Displayed in cells is n (%) for categorical parameter, mean (SD) for continuous parameter. Neer classification [4].

Parameter	All Patients
Age at surgery	51.6 (12)
Sex- Female	12 (71)
Sex- Male	5 (29)
Diabetes- No	15 (88)
Diabetes- Yes	2 (12)
Non-smoker	8 (47)
Smoker	9 (53)
Osteoporosis- No	15 (88)
Osteoporosis- Yes	2 (12)
Neer classification two-part	10 (59)
Neer classification three-part	5 (29)
Neer classification four-part	2 (12)

**Table 2:** Demographics of Follow-Up Patients Displayed in cells is n (%) for categorical parameter, mean (SD) for continuous parameter. Neer classification [4].

Parameter	Patients
Number of follow ups	14 (82)
Average follow-up duration (months)	4.7 (3.8)
Female	10 (71)
Male	4 (29)
Revision- No	12 (86)
Revision- Yes	2 (14)
Complications- No	13 (93)
Complications- Yes	1 (7)
Neer classification two-part	7 (50)
Neer classification three-part	5 (36)
Neer classification four-part	2 (14)

Functional outcomes in this cohort did not demonstrate clear patterns across sex, smoking status, or fracture classification, although the small sample size limits any definitive conclusions. Men and women reported comparable levels of shoulder function, disability, and satisfaction, with no clear pattern suggesting that sex influenced recovery. Smokers and nonsmokers also demonstrated similar functional profiles, indicating that smoking history did not appear to meaningfully affect early postoperative outcomes in this cohort.

When comparing outcomes across fracture types, patients with two, three, and four-part fractures all reported broadly similar ASES, DASH, and SANE scores [8-10]. Although patients with two-part fractures tended to report slightly better function and satisfaction, the overall pattern suggested that fracture complexity did not strongly influence patient-reported outcomes in the early postoperative period. These comparisons are detailed in Tables 3-5.

**Table 3:** Descriptive Statistics of Patient-Reported Functional Outcomes Grouped by Sex Displayed in cells is n (%) for categorical parameter, median (interquartile range) for continuous variables. p-value and Z-statistic were calculated using the Mann-Whitney U test comparing different outcome scores between male and female. ASES = American Shoulder and Elbow Society [8], DASH = Disabilities of the Arm, Shoulder and Hand [9], SANE = Single Assessment Numeric Evaluation [10].

Parameter	Female	Male	Z-statistic	p-value
ASES	83.3 (23.0)	97.3 (2.5)	1.026	0.30
DASH	11.6 (18.3)	1.3 (1.9)	-1.112	0.27
SANE	82.3 (20.5)	82 (21.7)	0	>0.99
Satisfaction Score	8.8 (2.6)	9.8 (0.5)	0.633	0.53

**Table 4:** Descriptive Statistics of Patient-Reported Functional Outcomes Grouped by Smoking Status Displayed in cells is n (%) for categorical parameter, median (interquartile range) for continuous variables. p-value and Z-statistic were calculated using the Mann-Whitney U test comparing different outcome scores between smokers and non-smokers. ASES = American Shoulder and Elbow Society [8], DASH = Disabilities of the Arm, Shoulder and Hand [9], SANE = Single Assessment Numeric Evaluation [10].

Parameter	Smoker	Non-Smoker	Z-statistic	p-value
ASES	89.0 (17.0)	85.6 (24.4)	0.197	0.84
DASH	8.0 (15.8)	9.3 (17.2)	-0.406	0.69
SANE	84.4 (20.7)	79.8 (20.6)	0.641	0.52
Satisfaction Score	9.63 (0.7)	8.5 (3.1)	-0.853	0.39

**Table 5:** Descriptive Statistics of Patient-Reported Functional Outcomes Grouped by Neer Classification Displayed in cells is n (%) for categorical variables, median (interquartile range) for continuous variables. p-value and H-statistic were calculated using the Kruskal-Wallis test to compare different outcome scores between two-part, three-part, and four-part fractures. ASES = American Shoulder and Elbow Society [8], DASH = Disabilities of the Arm, Shoulder and Hand [9], SANE = Single Assessment Numeric Evaluation [10]. Neer classification [4].

Parameter	Two-part	Three-part	Four-part	H-statistic	p-value
ASES	93.3 (16.0)	73.6 (26.9)	92.5 (3.5)	4.755	0.09
DASH	5.2 (15.2)	16.1 (19.9)	7.1 (4.1)	5.865	0.05
SANE	87.8 (20.3)	75 (22.4)	72.5 (3.5)	2.877	0.24
Satisfaction Score	9.8 (0.4)	7.6 (3.8)	9.5 (0.7)	2.856	0.24

Postoperative shoulder motion followed a similar trend. Abduction and forward flexion were comparable across all fracture groups, with no consistent differences indicating that more complex fractures resulted in worse early mobility. Despite the small sample size, these findings suggest that fracture classification alone may not be a strong predictor of early postoperative range of motion in younger patients treated with ORIF. Range of motion data are summarized in Table 6.

**Table 6:** Descriptive Statistics of Patient Range of Motion Grouped by Neer Classification Displayed in cells is n (%) for categorical variables, median (interquartile range) for continuous variables. p-value and H-statistic were calculated using the Kruskal-Wallis test comparing range of motion between two-part, three-part, and four-part fractures. Neer classification [4].

Parameter	Two-part	Three-part	Four-part	H-statistic	p-value
Abduction	105 (36.4)	100 (48.3)	90 (0)	0.164	0.92
Forward Flexion	114.3 (37.0)	111.3 (58.6)	90 (0)	0.236	0.89

## Discussion

PHFs present with distinct etiologies across different age groups. In elderly patients, these injuries typically result from low-energy falls, whereas in younger individuals, high-energy mechanisms are the predominant cause. There are many challenges in deciding the best method to treat PHFs. Common treatment options include ORIF, hemiarthroplasty (HA), and total/reverse shoulder arthroplasty (TSA/RTSA) [11]. In older populations, diminished bone quality is associated with higher reoperation rates and poorer outcomes following ORIF, leading to a greater reliance on arthroplasty [12-14]. For two- and three-part PHFs, ORIF typically yields a superior range of motion compared to HA, making it the preferred treatment for younger patients due to their higher functional demands and better bone quality [12]. Among available fixation techniques, the proximal humerus locking compression plate has gained widespread acceptance due to its fixed-angle construct, which enhances fracture stability [3,6,13].

Despite its frequent use, there is a notable gap in the literature regarding the clinical outcomes of this surgical construct specifically in the non-elderly population. Existing studies primarily emphasize elderly cohorts, leaving the applicability of current fixation strategies in younger patients less clearly defined. This study aims to address this limitation by evaluating the treatment efficacy and postoperative outcomes of ORIF for PHFs in non-elderly individuals.

Reoperation rates and postoperative complications

Although our study is limited by a small cohort and short‑term follow‑up, the complication rate we observed was nonetheless lower than that described in prior reports. ORIF is commonly associated with a high risk of complications, including avascular necrosis, infection, screw penetration, and varus collapse [13,15]. Despite these recognized risks, our findings indicate a comparatively lower incidence of postoperative complications. Notably, the two patients who required revision surgery were both females, with one sustaining a two-part fracture that led to screw pullout and the other experiencing nonunion following a three-part fracture. These findings highlight the variability in patient outcomes and suggest that fracture pattern and individual patient factors may contribute to postoperative complication rates.

Differences in functional outcome scores on patient factors: sex, smoking status, and fracture classification

Demographic analysis of our study cohort revealed a mean age of 51.6 and a predominance of female patients, aligning with existing literature indicating a higher incidence of PHFs in women aged 50 years and older compared to men [1,16]. While this trend is well established in elderly populations, where osteoporosis contributes significantly to fracture risk, it is somewhat unexpected in younger cohorts, given that high-energy trauma is more frequently observed in males [1]. This finding underscores the complex interplay between age-related bone quality and injury mechanisms, warranting further investigation into sex-specific fracture patterns and their implications for treatment strategies.

In our cohort, the most common fracture type was a two-part fracture pattern, followed by three-part and lastly four-part. Our study demonstrated that no statistically significant differences in functional outcomes were observed between smokers and non-smokers, suggesting that smoking status did not appear to influence postoperative recovery within the cohort studied.

Despite the need for revision in two patients, functional outcomes for two-, three-, and four-part fractures demonstrated overall favorable results. Comparative analysis of functional scores across fracture patterns revealed no significant differences in ASES, DASH, or SANE scores [8-10]. Patients with two-part fractures exhibited the lowest DASH scores, followed by those with four-part and three-part fractures. Similarly, ASES and SANE questionnaire surveys reflected positive functional recovery, with satisfaction scores indicating that patients were generally pleased with their outcomes. Notably, individuals with two-part fractures reported the highest satisfaction, followed by those with four-part and three-part fractures. This study contributes to the existing literature by providing additional insight into reported outcomes for two- and three-part fractures. Despite the limited sample size, our findings are consistent with those of Zhang et al., who evaluated three- and four-part fractures in young, non-osteoporotic patients and reported no significant differences in functional outcomes across fracture patterns. Similarly, George P. et al. concluded that younger patients experience superior functional outcomes and reduced complication rates [13,14].

Differences in range of motion between Neer fracture classifications

Notably, our findings revealed no statistically significant differences in postoperative range of motion across the various fracture types; this may suggest that fracture classification alone may not be a primary determinant of functional mobility outcomes in this cohort. Because only two patients in this study had four‑part fractures, and one of these individuals demonstrated persistent rotational deficits, the evidence is too limited to make a strong claim about the effectiveness of ORIF with locking plates for this fracture pattern. The potential viability of this approach for four‑part fractures remains speculative and requires further investigation. Given the complexity of these fractures, experienced surgeons should carefully evaluate individual patient factors to optimize surgical outcomes when performing ORIF in younger individuals.

Limitations

Using outcome measures to assess patient recovery following intervention is not without limitations. Some outcome scores focus on the objective aspects of injury and not the subjective aspects that are important to patients who experience restrictions and activity limitations following a PHF [17]. There are also a variety of outcome measures that can be used for evaluating clinical outcomes, and the literature has reported the lack of standardization in the outcome measures used for PHFs. Van de Water et al. identified 17 outcome measures that have been used in studies in patients with PHFs [18]. The lack of standardization has made it difficult to compare studies and for meta-analysis. In the study done by Zhang et al., the authors evaluated patient outcomes using the University of California Los Angeles (UCLA) score and the Constant score [13]. The Constant score has been reported to have inconsistent methodology across studies and lacks validity and reliability making it an unreliable score to use for assessing PHFs [17,19]. In our study, we opted to use the ASES, DASH and SANE scores [8-10]. The ASES and DASH scores consider the patient’s own perception of their symptoms, as well as their functional ability and disability in daily life [8,9]. The SANE score is another subjective assessment used to evaluate the patient’s own perception of function and has been reported to be a reliable and efficient assessment for shoulder pathologies [10]. Richard et al. reported using the DASH, ASES, and SANE scores as the minimum recommendation in shoulder-specific outcome measures when evaluating patients with PHFs in future studies [19]. We hope that by using these outcome measures we can capture the best representation of patient recovery from their perspective. Additionally, by using these recommended outcome measures, we hope to help address the lack of standardization in literature.

Additionally, as a retrospective study, direct comparisons between different PHF patterns were inherently limited, making equitable evaluation challenging. Due to limitations in patient follow-up, direct physical examinations could not be consistently obtained. Consequently, objective outcome data were derived from retrospective chart reviews, which may underestimate the true extent of suboptimal clinical outcomes. In our cohort, three patients were lost to follow-up and thus lacked final range of motion assessments. Loss of contact was attributed to outdated or incomplete contact information at the time of final evaluation. Loss to follow-up that is independent of health outcomes is generally associated with minimal risk of bias and is unlikely to influence the validity of our conclusions. Second, the absence of a suitable comparison group further restricted analysis, as few patients underwent operative treatment without locking plates during the study period, preventing an adequate comparative assessment. Furthermore, sample size expansion was constrained due to limitations in electronic medical record documentation prior to 2018. Third, the use of the revised Neer classification may have introduced interobserver variability, though this limitation was mitigated by the institutional patient volume over the study period. Despite these constraints, this cohort study contributes valuable insights to the currently limited literature on ORIF outcomes in non-elderly patients with PHFs. Future studies with larger prospective cohorts and enhanced statistical power should explore comparative outcomes between ORIF and alternative treatment modalities, including nonoperative management, hemiarthroplasty, total shoulder arthroplasty, and reverse shoulder arthroplasty. Despite extensive research, no single treatment has demonstrated superiority across all PHF types, and consensus remains limited. In one study, experienced shoulder surgeons agreed on an operative approach (HA, ORIF, or RSA) in only 50.9% of cases [20].

## Conclusions

In this study of non-elderly patients treated with locking plate fixation for PHFs, functional outcomes were generally favorable, complication and revision rates were low, and neither demographic factors nor fracture classification appeared to significantly influence early postoperative recovery. Patients across two-, three-, and four-part fracture patterns demonstrated comparable functional scores and range of motion, suggesting that younger individuals may achieve reliable outcomes regardless of fracture complexity when treated with ORIF. Although limited by sample size and follow-up duration, these findings provide meaningful insight into an understudied population and support locking plate fixation as a reasonable treatment option for PHFs in younger patients. Further research with larger cohorts is needed to refine predictors of success and guide treatment selection in this group.
